# Retraction Note: Taurine ameliorates thioacetamide induced liver fibrosis in rats via modulation of toll like receptor 4/nuclear factor kappa B signaling pathway

**DOI:** 10.1038/s41598-023-35116-5

**Published:** 2023-05-30

**Authors:** Nancy S. Younis, Amal M. H. Ghanim, Mohammad A. Elmorsy, Heba A. Metwaly

**Affiliations:** 1 Department of Pharmaceutical Sciences, College of Clinical Pharmacy, King Faisal University, Al-Ahsa, Kingdom of Saudi Arabia; 2 Department of Pharmacology, Zagazig University, Zagazig, Egypt; 3 Department of Biochemistry, Faculty of Pharmacy, Fayoum University, Fayoum, 63514 Egypt; 4 Department of Pharmaceutical Organic Chemistry, Faculty of Pharmacy, Mansoura University, Mansoura, Egypt; 5 Department of Pharmaceutical Chemistry, Faculty of Pharmacy, Delta University, Gamasa, 35712 Egypt; 6 Department of Biochemistry, Faculty of Pharmacy, Delta University, Gamasa, 35712 Egypt; 7 Department of Pharmaceutical Biochemistry, Faculty of Pharmacy, Alexandria University, Alexandria, 21500 Egypt

Retraction of: *Scientific Reports* 10.1038/s41598-021-91666-6, published online 10 June 2021

The Editors have retracted this Article. After publication of this Article it was brought to the Editors’ attention that some of the data in Figure 4A and 4I appear to overlap with data in Figure 1a and 1e published in [1] where it is partially attributed to a different sample. The second article was published by some of the same authors and was under consideration at the same time. The Editors reached out to the Authors to request raw data. The provided data did not resolve the concerns. Therefore the Editors no longer have confidence in the results described in this Article.

All Authors disagree with the retraction of the Article.
